# Boosting Alfalfa (*Medicago sativa* L.) Production With Rhizobacteria From Various Plants in Saudi Arabia

**DOI:** 10.3389/fmicb.2018.00477

**Published:** 2018-04-04

**Authors:** Ihsanullah Daur, Maged M. Saad, Abdul Aziz Eida, Shakeel Ahmad, Zahid Hussain Shah, Muhammad Z. Ihsan, Yasir Muhammad, Sayed S. Sohrab, Heribert Hirt

**Affiliations:** ^1^Department of Arid Land Agriculture, King Abdulaziz University, Jeddah, Saudi Arabia; ^2^Desert Agriculture Initiative, King Abdullah University of Science and Technology, Thuwal, Saudi Arabia; ^3^Agriculture Genetic Engineering Research Institute, Agriculture Research Center, Giza, Egypt; ^4^Department of Soil and Environmental Science, Muhammad Nawaz Shareef University of Agriculture, Multan, Pakistan; ^5^Cholistan Institute of Desert Studies, Islamia University Bahawalpur, Bahawalpur, Pakistan; ^6^King Fahd Medical Research Center, King Abdulaziz University, Jeddah, Saudi Arabia

**Keywords:** plant growth promoting rhizobacteria, abiotic stress, desert plants, sustainable agriculture, biofertilizer

## Abstract

This study focused on rhizobacteria to promote sustainable crop production in arid regions of Saudi Arabia. The study isolated 17 tightly root-adhering rhizobacteria from various plants at Hada Al Sham in Saudi Arabia. All 17 rhizobacterial isolates were confirmed as plant growth promoting rhizobacteria by classical biochemical tests. Using 16S rDNA gene sequence analyses, the strains were identified as *Bacillus, Acinetobacter* and *Enterobacter*. Subsequently, the strains were assessed for their ability to improve the physiology, nutrient uptake, growth, and yield of alfalfa plants grown under desert agriculture conditions. The field trials were conducted in a randomized complete block design. Inoculation of alfalfa with any of these 17 strains improved the relative water content; chlorophyll a; chlorophyll b; carotenoid contents; nitrogen (N), phosphorus, and potassium contents; plant height; leaf-to-stem ratio; and fresh and dry weight. *Acinetobacter pittii* JD-14 was most effective to increase fresh and dry weight of alfalfa by 41 and 34%, respectively, when compared to non-inoculated control plants. Nevertheless, all strains enhanced crop traits when compared to controls plants, indicating that these desert rhizobacterial strains could be used to develop an eco-friendly biofertilizer for alfalfa and possibly other crop plants to enhance sustainable production in arid regions.

## Introduction

In the Middle East, the Kingdom of Saudi Arabia started subsidy programs in the 1980s for developing the agricultural sector with the goal of achieving self-sufficiency in food security. Although the kingdom became self-sufficient in wheat, it came at the expense of severe underground water depletion, compelling the government to gradually decrease and terminate wheat production in 2016. This led some farmers to switch from wheat to alfalfa production, a preferred source of nutrients for animal feed in the kingdom. Yet, alfalfa is a crop that requires even higher levels of water and, thus, the kingdom plans to gradually terminate green fodder production by 2019. This series of events prompted the kingdom to fund various research projects which aim to exploit alternative innovative technologies to meet the domestic agricultural demands.

The use of beneficial microbes, such as bacteria and fungi, associated with the roots of plants offers a promising innovative solution for enhancing crop production in arid and semi-arid regions such as Saudi Arabia (Nadeem et al., 2014; Hanin et al., 2016). Bacteria living in the rhizosphere (a thin layer surrounding the roots) with the ability to promote plant growth and stress tolerance are termed plant growth-promoting rhizobacteria (PGPR) (Lugtenberg and Kamilova, 2009; de Zelicourt et al., 2013). PGPR can enhance plant growth and stress tolerance through various direct and indirect mechanisms (Glick, 2012; Kaushal and Wani, 2016; Timmusk et al., 2017). They can produce hormones and growth regulators, and/or modulate their levels in plants, including auxins or indole acetic acid (IAA), gibberellic acid, salicylic acid, abscisic acid and ethylene (Ahmed and Hasnain, 2014; Turan et al., 2014; Singh and Jha, 2016). PGPR can also act as biofertilizers, enhancing nutrient availability and acquisition by solubilization of inaccessible nutrients (e.g., phosphate) (Khan et al., 2013), fixation of nitrogen (Ardley, 2017; Sankhla et al., 2017), production of chelators (e.g., siderophores) for solubilization of minerals (e.g., Iron) (Palhares et al., 2015). Furthermore, PGPR have been reported to reduce the negative impacts of various stresses due to their effectiveness in many metabolic and physiological processes, such as the bioremediation of heavy metals (Liu et al., 2015; Seneviratne et al., 2015; Karthik et al., 2017; Oves et al., 2017), pesticide degradation/tolerance (Dubey and Fulekar, 2013; Tétard-Jones and Edwards, 2016) and drought and salinity tolerance (Daffonchio et al., 2015; Mahmood et al., 2016). For exemple, Khan et al. (2009b) strongly support PGPR as substitute to conventional methods for remediation of metal-poisoned soils—where it maintain soil fertility and enhances plant growth. Oves et al. (2013) has reported Pseudomonas strain that not only reduced chromium uptake in chickpea but at the same time enhanced bio-chemical features of the crop on chromium treated soil.

Understanding the role of PGPR in functional growth promotion, particularly in terms of PGP activities, such as plant growth and yield enhancement requires continuous efforts to identify and characterize new PGPR under field conditions (Khan et al., 2009a). However, plant-microbe interactions are very complex and can be highly variable due to variations in ecological conditions, soil physicochemical properties, host responses, and bacterial fitness and adaptation mechanisms (You et al., 2016; Zhou et al., 2016; Eida et al., 2017). Soussi et al. (2016) reported that the selection and recruitment of bacteria by plants is dependent on bio-pedo-agroclimatic factors in addition to the plant genotype, and that their diversity and functional redundancy plays an important role in supporting the plant's health and development under abiotic stresses. Very few ecological microbiome studies have been performed in Saudi Arabia (Yasir et al., 2015). Fierer et al. (2012) reported similarity in phylogenetic and functional diversity of microbial communities in soil rhizospheres of both hot and cold deserts. However, they found a surprising taxonomic diversity in desert soil when compared to other biomes. Nevertheless, rhizosphere microbes in semi-arid and arid regions have been demonstrated to enhance the production of crops such as acacia (Ardley, 2017; Sankhla et al., 2017), canola (Ahmad et al., 2016a,b), mung bean (Mahmood et al., 2016), cabbage (Turan et al., 2014), and wheat (Alamri and Mostafa, 2009).

Due to the global necessity in securing food availability, especially in the semi-arid regions where land mass can be exploited, the keen interest of the Kingdom of Saudi Arabia in increasing domestic alfalfa production, and the lack of studies on PGPR in the desert regions of Saudi Arabia, our work focused on identifying and exploring the potential of the local PGPR and their effects on alfalfa production. A number of PGPR strains were isolated, identified, and characterized for their PGP activities and effects on alfalfa production in field conditions. Several strains displayed promising results in a number of parameters, and one strain showed an exceptional performance for use in commercial applications.

## Materials and methods

### Sample collection and isolation of rhizobacteria

The PGPR were isolated from soil adhered to roots of various plants (*Setaria viridis, Cenchrus ciliaris, Panicum antidotale, Amaranthus viridis* and *Dichanthium annulatum*) at the Agricultural Research Station of King Abdulaziz University situated at Hada Al Sham (21°48′3″N, 39°43′25″E) at an altitude of 235 m above sea level and in arid climatic conditions (average temperature and rainfall given in Table 1). The rhizobacteria were isolated according to the dilution plate technique adopted from Baig et al. (2012). Briefly, plants were uprooted from the field with sufficient amount of soil and transferred to laboratory in polyethylene bags. Non-rhizosphere soil was removed by gentle shaking leaving behind only rhizosphere soil (strongly adhering to roots). Then, the tightly adhering rhizosphere soil was separated from the roots under aseptic conditions by gentle rubbing the surface of roots, and the soil was collected in a sterile petri dish. Finally, 10^−1^ soil dilution (suspension) was prepared by mixing 1 g soil into 10 mL sterilized PBS (phosphate-buffered saline). Further dilutions of soil (10^−2^-10^−8^) were prepared from 10^−1^ soil dilution using sterilized PBS by serial dilution technique. 100 μL of each dilution was plated on LB (Luria-Bertani) agar plates and incubated for 48 h at 28°C. Consequently, bacterial colonies were purified to 17 isolates on the basis of morphology, pigmentation, and growth rate. Cultures of purified bacterial isolates were stored in 35% glycerol (w/v) at −80°C.

**Table 1:** Mean monthly temperature (°C) and precipitation (mm) at the experimental site.

**Weather parameters**	**Month**											
	**Jan**	**Feb**	**Mar**	**Apr**	**May**	**Jun**	**Jul**	**Aug**	**Sep**	**Oct**	**Nov**	**Dec**
Min. T °C (2013)	14.6	16.9	18.6	20.8	22.8	24.9	26.1	27.6	25.4	23.2	23.1	18.4
Min. T °C (2014)	14.7	17.0	19.0	21.1	22.6	24.8	26.3	28.1	25.2	23.0	23.2	18.5
Long term min. T °C	15.6	18.1	19.2	21.0	23.2	24.0	26.1	27.0	25.2	23.5	22.1	19.0
Max. T °C (2013)	28.2	29.0	29.5	31.7	34.9	36.3	36.9	37.4	35.9	35.0	29.9	29.0
Max. T °C (2014)	28.1	28.9	29.1	32.1	34.7	36.1	37.1	37.3	36.2	35.2	30.0	28.5
Long term max. T °C	28.1	29.0	30.4	32.8	35.0	36.0	37.1	37.5	36.2	35.0	39.8	28.4
2013 Rainfall (mm)	2.60	3.00	1.80	–	–	–	–	–	1.50	–	8.40	20.00
2014 Rainfall (mm)	5.10	2.10	3.00	–	–	–	–	–	–	5.20	14.80	2.40
Long term Rainfall (mm)	4.50	1.18	1.20	0.50	–	–		–	–	0.89	3.20	3.30

### 16S rRNA identification and classification of rhizbacteria

Purified strains were revived on LB agar plates from which a single colony was used to inoculate 10 mL of LB medium and incubated for 16 h at 28°C and 220 rpm. The cells were then centrifuged at 12,000 × g and the pellet was used for genomic DNA extraction using a DNeasy blood and tissue kit (Qiagen, Germany) according to the manufacturer's instructions. The amplification of the 16S rRNA gene was amplified using PCR master mix (Promega, Madison, WI, USA) with bacterial universal primer sets 27F and 1492R (27F: 5′-AGA GTT TGA TCC TGG CTC AG-3′ and 1492R: 5′-TACGGYTACCTT GTTACGACT T-3′). PCR amplification of 16S rRNA genes was performed in a thermal cycler (Bio-Rad, Hercules, CA, USA) using the following PCR conditions: initial denaturation at 95°C for 1 min, followed by 30 cycles of denaturation (95°C for 90 s), annealing (55°C for 45 s), extension (72°C for 1.5 min), and a final extension step for 5 min at 72°C. Amplification was confirmed by analyzing PCR products on 1% agarose gel. PCR products were cleaned and purified from incorporated primers and extra dNTPs using ExoSAP-IT (Affymetrix, Santa Clara, CA, USA) and sequenced using an ABI 3730xl DNA Analyzer (Applied Biosystems, Foster City, CA, USA). Resolved 16S rRNA gene sequences were BLAST searched against the National Center for Biotechnology Information (NCBI) (http://www.ncbi.nlm.nih.gov) database (Altschul et al., 1997). Multiple alignment of the nucleotide sequences was performed with the program MUSCLE (Edgar, 2004). The phylogenetic tree was constructed by the Neighbor-Joining method (Saitou and Nei, 1987), based on the Kimura 2-parameter model (Kimura, 1980), with bootstrap analysis (1,000 replications) using the software MEGA (version 7) (Kumar et al., 2016).

### Biochemical characterization of rhizobacteria for plant growth-promoting activities

The 17 rhizobacterial isolates were characterized for the following plant growth-promoting activities such as phosphate solubilization and the production of auxins (IAA), ACC deaminase, siderophore and phosphatase, according to standard procedures as described previously (Khalid et al., 2004; Ahmad et al., 2008; Zaidi et al., 2009).

### Experimental design of field trials

Examining the potential of PGPR on physiology, nutrition, growth, and yield of alfalfa, was conducted over a 2-season field trial at the Agricultural Research Station of King Abdulaziz University. The experiment was planned according to a randomized complete block design (RCBD) with four replications (sub-plot sizes: 2.0 × 2.5 m).

Inoculation of alfalfa seeds (*Medicago sativa* L. cv. CUF-101) was performed by coating the seeds with a mixture containing broth culture of the individual PGPR strain (JD-1 to JD-17), sterilized peat, and sterilized sugar solution (10%) with a ratio of 4:5:1 v/v (Sajid et al., 2016). Alfalfa seeds were coated with the slurry at a rate of 50 mL kg^−1^, and control (non-inoculated) seeds were coated with a mixture containing all components without the bacterial strain. An organic fertilizer (30 tons ha^1^ composted cow manure) was applied and mixed with tractor in the experimental field every year, 15 days before sowing. At the time of sowing, 100 kg ha^−1^ NPK (20:20:20) was also added to the experimental field. The crop was sown each year at the beginning of October. As the crop was grown on previously inoculated alfalfa grown site, the seeds were not inoculated with Rhizobia.

Alfalfa, a perennial crop, is considered an important forage crop in Saudi Arabia as an animal feed. In climates with cold winters, alfalfa takes approximately 3 months to establish before producing its highest yields. The number of possible harvests/cuts varies with climate and ranges between 2 and 12 cuts per year. However, in climates similar to that of the Middle East, it is grown as an annual crop, giving 12 cuts per year, and can often be left for 3–4 years continuously. The first cut was performed 35 days after sowing, and subsequent cuts were carried out at 30-day intervals. Although alfalfa is a perennial crop, to test the PGPR efficiency the crop was grown separately in each season and discontinued after eight cuts. Cuttings were done during the early-bloom stage. All agronomic practices, except for the PGPR treatments, were conducted uniformly throughout field experimentation. For example, seeds were sown on both sides of drip irrigation lines at a distance of 10 cm, with 30 cm between drip lines. The crop was uniformly irrigated daily in the morning for 10 min using automatic control drip irrigation. Hand weeding was always practiced; the first weeding was done at 20 days after sowing and subsequent weeding were done 5 days after each cutting.

### Data collection and analysis

The soil texture was analyzed before the start of experiment, and the soil properties were analyzed prior to and after finishing the experiment, following the procedures of Ryan et al. (2001) and average values for both seasons are given in Table 2.

**Table 2:** Physicochemical properties of soil at the experimental site before and after the field trials.

**Feature (unit)**	**Initial values**	**Final values**
Sand (%)	75	
Silt (%)	14	
Clay (%)	11	
**Textural class**	**Sandy loam**	
pH	7.73 ± 0.01	7.69 ± 0.13
EC (dS m^−1^)	2.9 ± 0.14	3.0 ± 0.00
Organic matter (%)	0.63 ± 0.04	1.20 ± 0.57
Total Nitrogen (%)	0.037 ± 0.00	0.040 ± 0.00
Available Phosphorus (mg kg^−1^)	5.8 ± 0.42	5.2 ± 0.28
Extractable Potassium (mg kg^−1^)	95 ± 9.90	80 ± 2.83
Magnesium (mg kg^−1^)	184 ± 86.00	149 ± 44.00
Zinc (mg kg^−1^)	76 ± 5.66	53 ± 2.83
Iron (mg kg^−1^)	28 ± 2.83	16 ± 2.83
Copper (mg kg^−1^)	8 ± 1.41	6 ± 1.41
Manganese (mg kg^−1^)	10 ± 2.83	7 ± 1.41

*Data presented are means of 2 years (± standard deviation)*.

Assessment of PGPR effects on alfalfa crop performance was done by measuring the relative water content (RWC), photosynthetic pigments content, growth and yield parameters, and nutrient (nitrogen/N, phosphorous/P, and potassium/K; NPK) content at each cutting stage. The relative water content (RWC) measures the water content of leaf tissue relative to the maximal water content it can hold at full turgidity. The RWC was measured using fresh, fully developed leaves randomly selected from the tops of plants. After sampling, the leaves were transported to the laboratory in sealed plastic bags where fresh weights were measured immediately. Subsequently, the leaves were submerged in distilled water for 24 h. After that, the leaves were blotted with a paper towel, and then the fully turgid weight was recorded. Finally, the leaves were oven-dried at 72°C for 24 h and the dry weight was measured. The RWC was then determined according to the equation of Teulat et al. (2003):

$$\begin{array}{l} \text{RWC }\left(\%\right)=\;\frac{\text{Fresh weight}-\text{Dry weight}}{\text{Fully turgid weight}-\text{Dry weight}}\;\times 100 \end{array}$$

Chlorophyll measurements were done for fresh leaf samples using the protocol of Arnon (1949) with slight modifications. Briefly, 0.5 g from randomly selected leaves from each sub-plot was homogenized in a tissue homogenizer with 10 mL of 80% acetone. The homogenized sample mixture was centrifuged at 13,000 rpm for 15 min at 4°C. The supernatant was separated and used to measure absorbance for chlorophyll a (Chl a), chlorophyll b (Chl b), and total carotenoids at 663, 645, and 470 nm, respectively, using a spectrophotometer. Blank 80% acetone was used to zero the instrument initially and to reset it after every wavelength. Finally, the amounts of Chl a, Chl b and total carotenoids (C = x + c = xanthophylls and carotenes) were quantified (mg/mL) using the following equations.

$$\begin{array}{l} \text{Chla}=12.25\;{\text{A}}_{663}-2.79\;{\text{A}}_{645}\\ \text{Chlb}=21.5\;{\text{A}}_{645}-5.1\;{\text{A}}_{663}\\ \text{C x}+\text{c}=\left(1000\;{\text{A}}_{470}-1.82\text{ Chl a}-85.02\text{ Chl b}\right)/198 \end{array}$$

The chlorophyll amount was then converted to mg/g using the following equation:

$$\begin{array}{l} \text{Chl}=\left(\text{C x V}\right)/\left(1\text{ x g}\right)\\ \text{V}=\text{Volume of acetone used }\left(10\text{mL}\right)\\ \text{g}=\text{Weight of plant tissue used }\left(0.5\text{g}\right) \end{array}$$

### Crop growth, yield, and mineral analysis

Data on plant height (cm), leaf-to-stem ratio (LSR), and fresh and dry yield (kg ha^−1^), were recorded following Daur et al. (2011), Bakhashwain et al. (2013), and Ihsan et al. (2016). Accordingly, 5 plants from each sub-plot were randomly selected at harvesting time for measurement of plant height (cm). Fresh and dry weight yield were calculated based on the harvest of 4 central rows in each sub-plot. N, P, and K contents of the dry biomass of alfalfa were determined according to the protocol described by Bakhashwain et al. (2013). The N was determined by the Kjeldahl method, while P and K were determined by an inductively coupled plasma-optical emission spectrometer (ICP-OES, Varian 720-ES, Palo Alto, TX, USA). Furthermore, equally and effective nodulation was observed for the crop.

### Statistical analysis

The data collected on different parameters of alfalfa were analyzed using SAS/STAT software (https://www.sas.com/) and the means were compared using Fisher's least significant differences (LSD) test (Williams and Abdi, 2010).

### Accession numbers

The 16S rRNA gene sequences of the bacterial isolates in this study have been deposited at DDBJ/EMBL/GeneBank under accession numbers (KY941113 – KY941129).

## Results

### Isolation and identification of rhizobacteria

**Figure 1:**
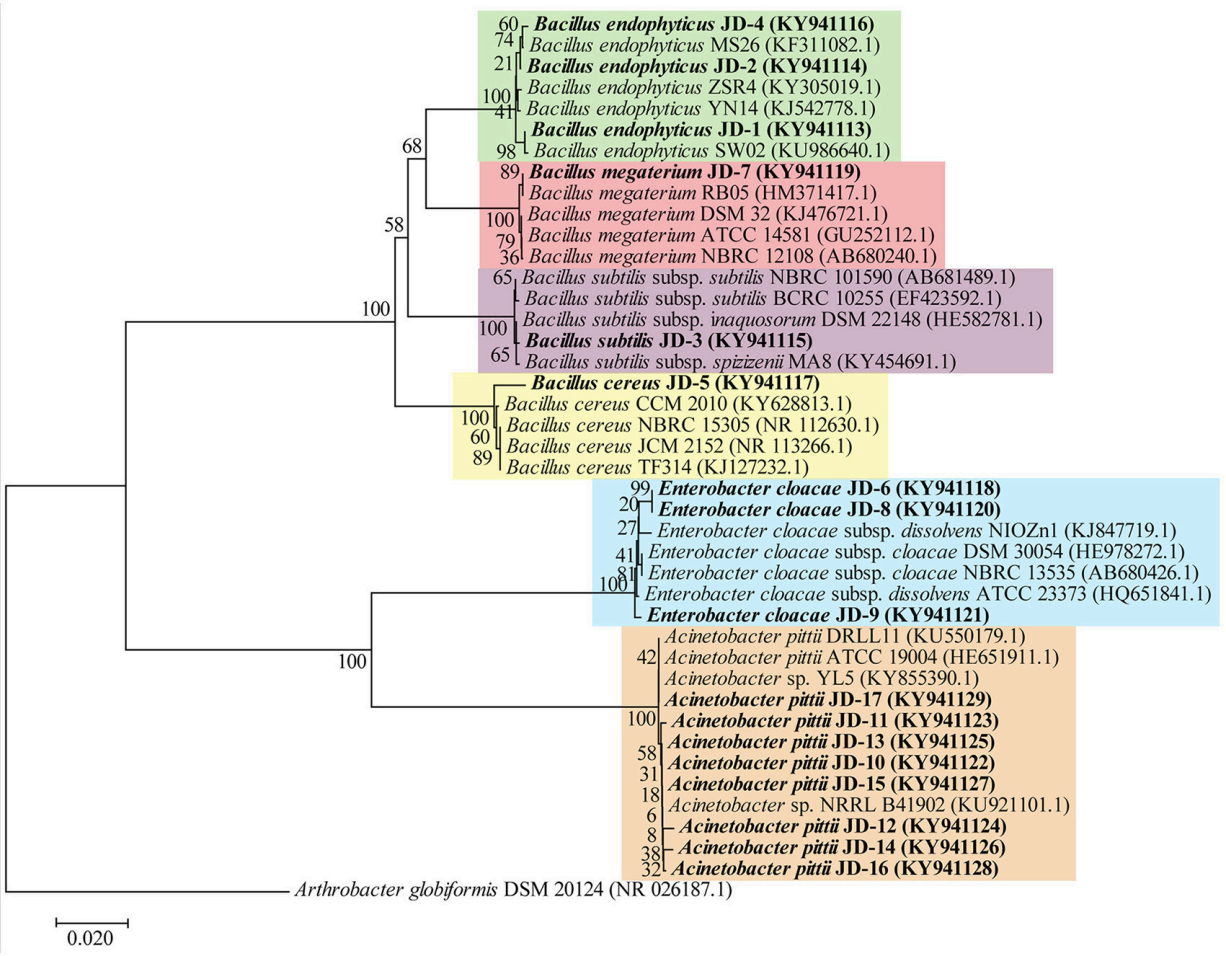
Phylogenetic tree of rhizosphere bacteria based on 16S rRNA gene sequence comparison. Evolutionary relationships of the bacterial strains (in bold) inferred using the Neighbor-Joining method and the evolutionary distances were computed using the Kimura 2-parameter method. GenBank accession numbers of strains are presented in parentheses. The percentage of replicate trees in which the associated taxa clustered together in the bootstrap test (1,000 replicates) are shown next to the branches.

In this study, 17 potential PGPR strains (JD-1 to JD-17) were isolated from the rhizosphere of various plant species growing at the King Abdulaziz University's Agricultural Research Station (Hada Al Sham, Kingdom of Saudi Arabia). Based on the 16S phylogenetic classification, the isolates were found to belong to two major phyla (Firmicutes and Proteobacteria), and were highly aligned with the genera *Bacillus, Enterobacter*, and *Acinetobacter* (Table 3). Alignment of the 17 strains with closely related strain sequences from the NCBI BLAST revealed six major species; *B. endophyticus, B. subtilis, B. megaterium, B. cereus, E. cloacae*, and *A. pittii* species (Figure 1). Although the *Bacillus* strains were diverse at the species level, the strains belonging to *Enterobacter* and *Acinetobacter* clustered together and, thus, are quite similar.

**Table 3:** List of rhizosphere bacteria isolated from Hada Al Sham and the top hits obtained from BLAST searches against the NCBI nucleotide database.

**Isolate**	**Phylum; Class; Order; Family**	**Plant species**	**Submitted name (GenBank ID)**	**Description**			
				**Top blast strains**	**Query cover (%)**	**Identity (%)**	**Accession**
JD-1	Firmicutes; Bacilli; Bacillales; Bacillaceae	*Setaria viridis*	*Bacillus endophyticus* JD-1 (KY941113)	*Bacillus endophyticus* 2DT	100	99	
JD-2	Firmicutes; Bacilli; Bacillales; Bacillaceae	*Setaria viridis*	*Bacillus endophyticus* JD-2 (KY941114)	*Bacillus endophyticus* 2DT	99	99	
JD-3	Firmicutes; Bacilli; Bacillales; Bacillaceae	*Setaria viridis*	*Bacillus subtilis* JD-3 (KY941115)	*Bacillus subtilis* subsp. inaquosorum BGSC 3A28	100	99	
JD-4	Firmicutes; Bacilli; Bacillales; Bacillaceae	*Setaria viridis*	*Bacillus endophyticus* JD-4 (KY941116)	*Bacillus endophyticus* 2DT	100	99	
JD-5	Firmicutes; Bacilli; Bacillales; Bacillaceae	*Cenchrus ciliaris*	*Bacillus cereus* JD-5 (KY941117)	*Bacillus cereus* CCM 2010	100	99	
JD-6	Proteobacteria; Gammaproteobacteria; Enterobacteriales; Enterbacteriaceae	*Cenchrus ciliaris*	*Enterobacter cloacae* JD-6 (KY941118)	*Enterobacter cloacae* subsp. cloacae ATCC 13047	100	99	
JD-7	Firmicutes; Bacilli; Bacillales; Bacillaceae	*Cenchrus ciliaris*	*Bacillus megaterium* JD-7 (KY941119)	*Bacillus megaterium* NBRC 15308	100	99	
JD-8	Proteobacteria; Gammaproteobacteria; Enterobacteriales; Enterbacteriaceae	*Panicum antidotale*	*Enterobacter cloacae* JD-8 (KY941120)	*Enterobacter cloacae* subsp. cloacae ATCC 13047	100	99	
JD-9	Proteobacteria; Gammaproteobacteria; Enterobacteriales; Enterbacteriaceae	*Panicum antidotale*	*Enterobacter cloacae* JD-9 (KY941121)	*Enterobacter cloacae* subsp. dissolvens LMG 2683	100	99	
JD-10	Proteobacteria; Gammaproteobacteria; Pseudomonadales; Moraxellaceae	*Panicum antidotale*	*Acinetobacter pittii* JD-10 (KY941122)	*Acinetobacter* sp. NRRL B-41902	100	99	
JD-11	Proteobacteria; Gammaproteobacteria; Pseudomonadales; Moraxellaceae	*Amaranthus viridis*	*Acinetobacter pittii* JD-11 (KY941123)	*Acinetobacter pittii* ATCC 19004	100	99	
JD-12	Proteobacteria; Gammaproteobacteria; Pseudomonadales; Moraxellaceae	*Amaranthus viridis*	*Acinetobacter pittii* JD-12 (KY941124)	*Acinetobacter pittii* ATCC 19004	100	99	
JD-13	Proteobacteria; Gammaproteobacteria; Pseudomonadales; Moraxellaceae	*Amaranthus viridis*	*Acinetobacter pittii* JD-13 (KY941125)	*Acinetobacter* sp. NRRL B-41902	100	99	
JD-14	Proteobacteria; Gammaproteobacteria; Pseudomonadales; Moraxellaceae	*Dichanthium annulatum*	*Acinetobacter pittii* JD-14 (KY941126)	*Acinetobacter* sp. NRRL B-41902	99	99	
JD-15	Proteobacteria; Gammaproteobacteria; Pseudomonadales; Moraxellaceae	*Dichanthium annulatum*	*Acinetobacter pittii* JD-15 (KY941127)	*Acinetobacter pittii* ATCC 19004	100	99	
JD-16	Proteobacteria; Gammaproteobacteria; Pseudomonadales; Moraxellaceae	*Dichanthium annulatum*	*Acinetobacter pittii* JD-16 (KY941128)	*Acinetobacter pittii* ATCC 19004	100	99	
JD-17	Proteobacteria; Gammaproteobacteria; Pseudomonadales; Moraxellaceae	*Dichanthium annulatum*	*Acinetobacter pittii* JD-17 (KY941129)	*Acinetobacter pittii* ATCC 19004	100	99	

### Characterization of rhizobacteria

The biochemical characterization of the isolates confirmed their effectiveness as plant growth-promoting microbes (Table 4). Strain JD-15 produced the highest amount of IAA (4.67 μg mL^−1^) with tryptophan, while strain JD-17 produced the highest (2.21 μg mL^−1^) without tryptophan. Phosphate solubility was highest for strain JD-9 (28.57 mg mL^−1^) compared to other strains. The highest recorded ACC-deaminase activity was for strain JD-14 (51 μM α-ketobutyrate mg^−1^ h^−1^) compared to all other strains. Siderophore and phosphatase production abilities were also variable. JD-14 and 10 other strains scored positive for siderophore production, phosphatase activity, or both. The biochemical characterization results indicated that all strains possessed some PGP activity. However, PGP assays are not sufficient as an indicator for a successful PGPR in the field. Therefore, all 17 PGPR strains were tested with alfalfa under field conditions.

**Table 4:** Biochemical characterization of rhizobacteria for plant growth-promoting activities.

**Strain**	**IAA production (**μ**g mL**^**−1**^**)**		**Phosphatesolubilization(mg P mL^−1^)**	**ACC-deaminaseactivity (μMα-ketobutyrate mg^−1^ h^−1^)**	**Siderophoreproduction**	**Phosphataseproduction**
	**With tryptophan**	**Without tryptophan**				
JD-1	1.21	ND^*^	5.52	7	–	+
JD-2	1.73	ND	11.34	ND	–	–
JD-3	1.11	0.84	12.56	ND	+	–
JD-4	3.73	1.79	19.15	15	–	+
JD-5	2.43	ND	7.57	5	+	+
JD-6	2.86	0.59	5.24	ND	–	+
JD-7	1.71	ND	5.23	10	+	–
JD-8	1.98	ND	4.45	4	–	–
JD-9	3.41	1.24	28.57	ND	–	+
JD-10	2.25	1.11	7.56	ND	–	–
JD-11	3.32	1.23	3.98	ND	–	–
JD-12	2.67	0.97	5.12	12	–	–
JD-13	3.54	1.32	8.43	ND	+	–
JD-14	1.51	ND	9.32	51	+	+
JD-15	4.67	ND	7.24	ND	–	–
JD-16	2.53	0.86	21.16	7	+	+
JD-17	1.17	2.21	5.25	14	+	–

* *ND, not detected, +, +ve for activity, –, no activity found*.

### RWC and photosynthetic pigments

Significant differences (*P* < 0.05) between PGPR-inoculated and non-inoculated control were observed for the RWC and photosynthetic pigments (Chl a, Chl b, and carotenoids) (Table 5). The highest RWC was observed when strains JD-14 (97.9%) and JD-2 (97.6%) were inoculated with alfalfa, while the lowest value was noted for the non-inoculated control plants (79.1%) All PGPR strains increased photosynthetic pigment content in alfalfa plants. However, strain JD-14 was the most effective strain in increasing Chl a (2.75 mg g^−1^) and Chl b (1.75 mg g^−1^), compared to non-inoculated control plants which contained 2.32 mg g^−1^ of Chl a and 1.06 mg g^−1^ of Chl b. Carotenoid content was found to be highest in non-inoculated control plants (0.92 mg g^−1^), while it was lowest in plants inoculated with strains JD-4 and JD-5 (0.71 mg g^−1^).

**Table 5:** Alfalfa relative water content (RWC) and contents of chlorophyll (Chl a, Chl b) and carotenoid (Car).

**Strain**	**RWC (%)**	**Chl a (mg g^−1^)**	**Chl b (mg g^−1^)**	**Car (mg g^−1^)**
Control	79.1 ± 0.99^h^	2.32 ± 0.01^k^	1.06 ± 0.01^k^	0.92 ± 0.02^a^
JD-1	93.8 ± 1.67^abc^	2.54 ± 0.02^bc^	1.28 ± 0.02^c−g^	0.72 ± 0.03^de^
JD-2	97.6 ± 1.39^ab^	2.53 ± 0.01^bc^	1.30 ± 0.01^cde^	0.74 ± 0.01^de^
JD-3	93.5 ± 2.79^abc^	2.51 ± 0.01^bcd^	1.29 ± 0.01^c−f^	0.74 ± 0.01^de^
JD-4	92.9 ± 2.50^bcd^	2.46 ± 0.03^def^	1.21 ± 0.02^g−j^	0.71 ± 0.02^e^
JD-5	90.2 ± 0.47^c−f^	2.46 ± 0.02^def^	1.21 ± 0.01^g−j^	0.71 ± 0.01^e^
JD-6	91.2 ± 1.23^cde^	2.55 ± 0.02^b^	1.36 ± 0.02^bc^	0.78 ± 0.04^cde^
JD-7	93.3 ± 2.96^abc^	2.49 ± 0.02^cde^	1.26 ± 0.02^e−h^	0.74 ± 0.01^de^
JD-8	88.2 ± 1.12^d−g^	2.54 ± 0.02^bc^	1.35 ± 0.02^bcd^	0.86 ± 0.05^abc^
JD-9	91.3 ± 2.64^cde^	2.45 ± 0.01^e−h^	1.23 ± 0.02^e−i^	0.81 ± 0.06^bcd^
JD-10	90.9 ± 1.74^c−f^	2.40 ± 0.02^ij^	1.14 ± 0.04^j^	0.88 ± 0.01^ab^
JD-11	86.2 ± 1.69^gf^	2.40 ± 0.01^hij^	1.16 ± 0.03^ij^	0.89 ± 0.01^ab^
JD-12	86.1 ± 2.95^gf^	2.41 ± 0.01^g−j^	1.17 ± 0.03^hij^	0.89 ± 0.01^ab^
JD-13	90.2 ± 0.99^c−f^	2.46 ± 0.01^d−g^	1.27 ± 0.02^d−g^	0.88 ± 0.04^ab^
JD-14	97.9 ± 1.23^a^	2.75 ± 0.02^a^	1.75 ± 0.05^a^	0.78 ± 0.06^cde^
JD-15	87.4 ± 1.52^gef^	2.44 ± 0.01^e−i^	1.22 ± 0.01^e−j^	0.91 ± 0.01^a^
JD-16	92.6 ± 1.00^dc^	2.56 ± 0.01^b^	1.40 ± 0.02^b^	0.91 ± 0.01^a^
JD-17	84.5 ± 0.99^g^	2.42 ± 0.01^f−j^	1.21 ± 0.04^f−j^	0.90 ± 0.01^a^

Data presented are means of three repeats over 2 years (± standard deviation). Mean^*^ sharing similar letters are not significantly different at P < 0.05 according to LSD.

### Mineral contents

Mineral contents, including N, P, K, and Mg, were significantly (*P* < 0.05) affected by the PGPR treatments (Table 6). Marked increases in N, P, K, and Mg contents were noted for the PGPR treatments compared to the non-inoculated control. Among the inoculated treatments, N content ranged from 37.7 to 49.5 mg g^−1^, P from 4.13 to 5.55 mg g^−1^, K from 23.67 to 29.78 mg g^−1^, and Mg from 2.45 to 2.90 mg g^−1^. Interestingly, the highest N and Mg contents were recorded for strain JD-14, while the highest P and K content were for strain JD-9. The lowest amounts of N (30.7 mg g^−1^), P (3.2 mg g^−1^), and Mg (2.31 mg g^1^) were recorded for the non-inoculated control treatment.

**Table 6:** Alfalfa nitrogen (N), phosphorous (P), potassium (K), and magnesium (Mg) nutrient content.

**Strain**	**N (mg g^−1^)**	**P (mg g^−1^)**	**K (mg g^−1^)**	**Mg (mg g^−1^)**
Control	30.7 ± 0.99^f^	3.20 ± 0.12^f^	20.38 ± 0.49^g^	2.31 ± 0.01^g^
JD-1	45.5 ± 1.66^abc^	5.05 ± 0.20^a^	27.76 ± 0.83^ab^	2.66 ± 0.02^bcd^
JD-2	45.3 ± 0.83^abc^	5.20 ± 0.15^a^	27.69 ± 0.41^ab^	2.67 ± 0.00^bc^
JD-3	45.1 ± 2.79^abc^	5.00 ± 0.34^ab^	27.58 ± 1.39^ab^	2.63 ± 0.02^bcd^
JD-4	44.5 ± 2.50^bcd^	4.93 ± 0.31^a−d^	27.27 ± 1.25^b−e^	2.58 ± 0.05^de^
JD-5	45.3 ± 0.99^abc^	4.96 ± 0.12^abc^	27.42 ± 0.48^a−d^	2.59 ± 0.03^cde^
JD-6	45.8 ± 1.11^abc^	5.46 ± 0.31^a^	27.90 ± 0.55^ab^	2.71 ± 0.03^b^
JD-7	44.9 ± 2.96^abc^	5.23 ± 0.31^a^	27.48 ± 1.48^abc^	2.63 ± 0.04^bcd^
JD-8	49.1 ± 1.16^ab^	5.40 ± 0.31^a^	29.54 ± 0.58^ab^	2.71 ± 0.03^b^
JD-9	46.7 ± 1.73^ab^	5.55 ± 0.15^a^	29.78 ± 0.61^a^	2.68 ± 0.01^b^
JD-10	39.9 ± 0.44^de^	4.30 ± 0.07^de^	24.77 ± 0.31^f^	2.47 ± 0.03^f^
JD-11	38.6 ± 1.36^e^	4.08 ± 0.21^e^	23.90 ± 0.84^f^	2.45 ± 0.03^f^
JD-12	37.7 ± 2.95^e^	4.08 ± 0.36^e^	23.87 ± 1.47^f^	2.46 ± 0.02^f^
JD-13	40.0 ± 0.29^de^	4.36 ± 0.03^b−e^	25.00 ± 0.14^def^	2.53 ± 0.01^ef^
JD-14	49.5 ± 1.23^a^	5.23 ± 0.14^a^	28.38 ± 0.86^ab^	2.90 ± 0.03^a^
JD-15	41.6 ± 0.71^cde^	4.24 ± 0.19^e^	24.53 ± 0.76^f^	2.51 ± 0.02^ef^
JD-16	39.8 ± 1.12^e^	4.34 ± 0.14^cde^	24.94 ± 0.56^ef^	2.53 ± 0.02^ef^
JD-17	40.1 ± 1.83^de^	4.13 ± 0.31^e^	24.08 ± 1.27^f^	2.48 ± 0.03^f^

Data presented are means of three repeats over 2 years (± standard deviation). Mean^*^ sharing similar letters are not significantly different at P < 0.05 according to LSD.

### Crop growth and yield

PGPR considerably increased plant height, LSR, and fresh and dry weight yield of alfalfa plants compared to the non-inoculated control (Figure S1, Table 7). Plant height for PGPR inoculants ranged between 55.5 and 67.3 cm. The maximum plant height was noted for plants inoculated with strain JD-14 (67.3 cm), while the lowest plant height was noted for non-inoculated plants (48.5 cm). LSR ranged from 1.31 to 1.87 among strains, where strain JD-11 and JD-12 had a LSR of 1.31, strain JD-14 had an LSR of 1.87, and control plants had 1.24. Fresh weight yield ranged between 65.4 and 80.2 tons ha^−1^ among the strains. The highest fresh yield (80.2 tons ha^−1^) was recorded for strain JD-14, while the lowest fresh yield (56.7 tons ha^−1^) was noted for non-inoculated control plants.

**Table 7:** Alfalfa plant height (PH), leaf to stem ratio (LSR), fresh yield (FY) and dry yield (DY).

**Strain**	**PH (cm)**	**LSR**	**FY (tons ha^−1^)**	**DY (tons ha^−1^)**
Control	48.5 ± 0.99^g^	1.24 ± 0.06^d^	56.7 ± 1.24^f^	19.7 ± 0.53^f^
JD-1	63.3 ± 1.66^ab^	1.73 ± 0.07^ab^	75.1 ± 2.08^a^	24.8 ± 0.68^a^
JD-2	63.2 ± 0.84^ab^	1.74 ± 0.03^ab^	76.7 ± 1.54^a^	25.3 ± 0.51^a^
JD-3	62.9 ± 2.79^ab^	1.75 ± 0.02^ab^	74.7 ± 3.49^ab^	24.6 ± 1.15^ab^
JD-4	62.3 ± 2.50^b−e^	1.70 ± 0.02^b^	73.9 ± 3.13^a−d^	24.4 ± 1.03^a−d^
JD-5	62.6 ± 0.97^a−d^	1.69 ± 0.02^b^	74.3 ± 1.21^abc^	24.5 ± 0.40^abc^
JD-6	63.6 ± 1.11^ab^	1.76 ± 0.08^ab^	79.2 ± 3.12^a^	26.1 ± 1.03^a^
JD-7	62.7 ± 2.96^abc^	1.69 ± 0.03^b^	76.9 ± 3.11^a^	25.4 ± 1.02^a^
JD-8	66.9 ± 1.16^ab^	1.76 ± 0.04^ab^	78.6 ± 3.12^a^	25.9 ± 1.03^a^
JD-9	64.5 ± 1.74^ab^	1.79 ± 0.06^ab^	76.9 ± 1.42^a^	25.4 ± 0.47^a^
JD-10	57.3 ± 0.62^f^	1.40 ± 0.12^c^	67.6 ± 0.77^de^	22.3 ± 0.25^de^
JD-11	55.6 ± 1.69^f^	1.31 ± 0.02^cd^	65.5 ± 2.11^e^	22.3 ± 0.61^e^
JD-12	55.5 ± 2.95^f^	1.31 ± 0.02^cd^	65.4 ± 3.68^e^	22.2 ± 0.97^e^
JD-13	57.8 ± 0.29^def^	1.45 ± 0.10^c^	68.2 ± 0.36^b−e^	22.5 ± 0.12^cde^
JD-14	67.3 ± 1.23^a^	1.87 ± 0.04^a^	80.2 ± 1.54^a^	26.4 ± 0.51^a^
JD-15	56.8 ± 1.52^f^	1.34 ± 0.01^cd^	67.0 ± 1.90^e^	22.1 ± 0.62^e^
JD-16	57.6 ± 1.12^ef^	1.35 ± 0.01^cd^	68.1 ± 1.40^cde^	22.6 ± 0.35^b−e^
JD-17	55.9 ± 2.54^f^	1.33 ± 0.03^cd^	65.9 ± 3.17^e^	22.1 ± 1.09^e^

Data presented are means of three repeats over 2 years (± standard deviation). Mean^*^ sharing similar letters are not significantly different at P < 0.05 according to LSD.

## Discussion

In this study, 17 PGPR strains were isolated from desert plants located at the western desert region of Saudi Arabia. Based on 16S rRNA gene sequence analysis, the isolates were identified to belong to different species of *Bacillus, Enterobacter* and *Acinetobacter*. Species belonging to the same genera have been previously isolated, and their PGP abilities have been demonstrated. For example, *B. laevolacticus* and *B. amyloliquefaciens* isolated from the rhizosphere of cotton grown in the semi-arid region of Uzbekistan have been shown to increase plant growth of wheat and maize in pot experiments (Egamberdiyeva, 2005). *B. megaterium* isolated from the rhizosphere has been shown to promote the growth and alter the root system architecture of *Arabidopsis thaliana* model plants (López-Bucio et al., 2007). *E. cloacae* MSR1, isolated from roots of non-nodulating alfalfa plants at farms of Al-Ahsaa city in Saudi Arabia were shown to possess a number of PGP activities (Khalifa et al., 2016). Recently, a complete genome sequence analysis of an Enterobacter species, isolated from the Jizan region in Saudi Arabia, with multi-stress tolerance promoting activities has been published (Andrés-Barrao et al., 2017). Furthermore, *Acinetobacter* species were isolated from the rhizosphere of pearl millet in India and have presented PGP activities and abilities (Rokhbakhsh-Zamin et al., 2011). All strains showed a potential for IAA production, phosphate solubilization, ACC deaminase activity, and siderophore and phosphatase production in reasonable agreement with strain variations found in other studies (Hayat et al., 2013; Habibi et al., 2014; Zahid et al., 2015). Zhang et al. (2011) further supports our findings by reporting variability in ACC deaminase activity, siderophore production, and phosphate solubilization for 8 different rhizobacterial strains.

Given that the PGPR strains displayed a potential for PGP activities, their effects on alfalfa under the natural, arid field conditions of Saudi Arabia were tested. The relative water content (RWC) is one of the most common measures of plant water status in the scope of physiological consequences of water shortage in cells (Spomer, 1985). For example, wheat cultivars with high RWC were shown to be more resistant to drought stress (Schonfeld et al., 1988). In our study, plants inoculated with PGPR strains contained higher RWC, up to 18.8% (for strain JD-14), than non-inoculated control plants. This is an indication of enhanced water uptake due to bacterial inoculation, which generally helps in the development of a more effective root system, that can absorb more water from deep soil under stress conditions (Marulanda et al., 2010). Likewise, the increased RWC might indirectly or directly contribute to the increase in photosynthetic pigments (Chl a, Chl b, and carotenoid content) of inoculated alfalfa, in agreement with Ma et al. (2016). The enhanced production of photosynthetic pigments with microbial inoculation has previously been reported for various crops such as canola (Farshidi et al., 2012), wheat (Tuna et al., 2008; Chen et al., 2014), tomato (Haghighi and Pessarakli, 2013), and soybean (Lee et al., 2010). The increased chlorophyll content in plants results in an increased photosynthetic rate, converting more carbon dioxide and water to glucose, that boosts the metabolism and eventually increases plant growth under stress conditions (Kang et al., 2014).

Compared to control plants, PGPR inoculation increased alfalfa nutrient (N, P, K, and Mg) contents. There are various mechanisms for enhanced plant nutrition, such as nutrient availability, improved nutrient uptake and healthier root growth. PGPR may increase nutrient availability through solubilization of nutrients by releasing regular organic acids (Vyas and Gulati, 2009). PGPR are also able to produce enzymes, such as phytases and phosphatases, that play roles in releasing nutrients from soil organic matter and, thus, enhancing nutrient availability, as supported by Jorquera et al. (2008). In this study, variable *in vitro* solubilization of phosphate was observed with PGPR, which may indicate their potential to increase nutrient availability. Similarly, Calvo et al. (2014) suggested that better root growth and architecture observed with PGPR inoculation is a vital characteristic for improved nutrition. Studies already conducted to this end, such as the trials of Baig et al. (2012) and Shahzad et al. (2013), recorded improved root growth for plants inoculated with PGPR than for non-inoculated control plants. Furthermore, enhanced root growth and improved nutrient uptake with PGPR has been reported in many previous studies in various crops such as cabbage and tea (Vacheron et al., 2013; Turan et al., 2014; Çakmakçi, 2016). The superiority of strain JD-14 (*Acinetobacter pittii*) in this study may be due to increased phosphate solubilization and ACC deaminase activities, as our findings agree with those of Baig et al. (2012), which reported better nutrient uptake for PGPR that displayed phosphate solubilization and ACC deaminase activities than for those that did not. Likewise, Ahmad et al. (2016a), Subhashini et al. (2016), and Verma et al. (2016) reported enhanced nutrient content and uptake in different crops with PGPR application.

Plant height, LSR, and fresh and dry weight yields were remarkably improved with PGPR inoculation under field conditions. Previously, Naveed et al. (2014) reported an increase in leaf area for PGPR treatment under stress conditions. The ability of PGPR strains to produce and/or modulate plant hormones stimulated cell division and cell elongation which correlated with plant growth (Fahad et al., 2015; Verbon and Liberman, 2016). However, the qualitative PGPR capacity varied for each strain. In this work, IAA production was variable for the 17 PGPR strains. Moreover, as mentioned above, PGPR enhance nutrient availability and uptake, which according to Pii et al. (2015) and Metay et al. (2015), significantly promotes vegetative growth. Sarwar et al. (2016) considers better phosphate acquisition a key cause of enhanced plant growth and crop development. Additionally, improved yield attributes, such as increased plant fresh and dry weight, caused by PGPR inoculation might be due to improved physiological and metabolic processes, better nutrient uptake, and ultimately production of more photosynthate.

Some strains of *Bacillus cereus, Enterobacter cloacae* and *Acinetobacter pittii* were previously reported as opportunistic pathogens (Al Atrouni et al., 2016). Nevertheless, the 17 isolated strains in this study are ubiquitously distributed in the ecosystem (Jha et al., 2011; Mwashasha et al., 2016). *Acinetobacter pittii* is considered a normal habitant of human skin. *Enterobacter cloacae* is a member of the gut microbiota and has been previously reported as PGPR (Jha et al., 2011). *Bacillus cereus* is commonly found in soil and food, and only a few strains of *Bacillus cereus* are harmful, while others have been used as probiotics for animals (Li et al., 2015). Therefore, in general, these isolates should not be a risk factor but further studies including a screen for virulence factors might be needed before developing them for commercial applications.

In conclusion, the present study assessed the effects of PGPR on the physiology, nutrient uptake, growth, and yield of alfalfa. We revealed that strain JD-14 (*Acinetobacter pittii*) was the most effective strain in improving RWC, photosynthetic pigments, growth, and yield of alfalfa under arid conditions. However, all strains (JD-1 to JD-14) were found to be at least somewhat effective in enhancing alfalfa yield. Consequently, these findings suggest that PGPR application in agriculture is a sustainable strategy for increasing crop production, especially in arid regions. Although current programs for the development of the agricultural sector in Saudi Arabia foresee to gradually terminate green fodder production by 2019, a completely import-based food and feed supply is a risky economic strategy. Therefore, exploiting various technologies to meet the needs of the domestic agriculture is an important goal. Moreover, the results of the present experiments should be applicable to many countries with arid and semi-arid climates. Further studies are necessary to explore the influence of PGPR on the plant biochemistry and to assess the use of PGPR as a general strategy for improving yield of crops.

## Author contributions

ID, SA, and YM designed and executed the experiments. ID, ZS, MI, and SA collected data in the field and carried out the lab work regarding relative water content, chlorophyll measurement and nutrient analysis. ID, YM, SA performed the isolation and characterization, and MS and AE performed the identification of the PGPR. ID, MS, and SS wrote the manuscript. HH provided technical support and helped in writing the manuscript.

### Conflict of interest statement

The authors declare that the research was conducted in the absence of any commercial or financial relationships that could be construed as a potential conflict of interest. The reviewer MO declared a shared affiliation with no collaboration, with several of the authors ID, SA, and ZS to the handling Editor.
