# Supplementary figures and images for: Boosting Alfalfa (Medicago sativa L.) Production With Rhizobacteria From Various Plants in Saudi Arabia
Source: Front Microbiol. 2018 Apr 4;9:477. doi: 10.3389/fmicb.2018.00477 (PMC5893776; doi:10.3389/fmicb.2018.00477)

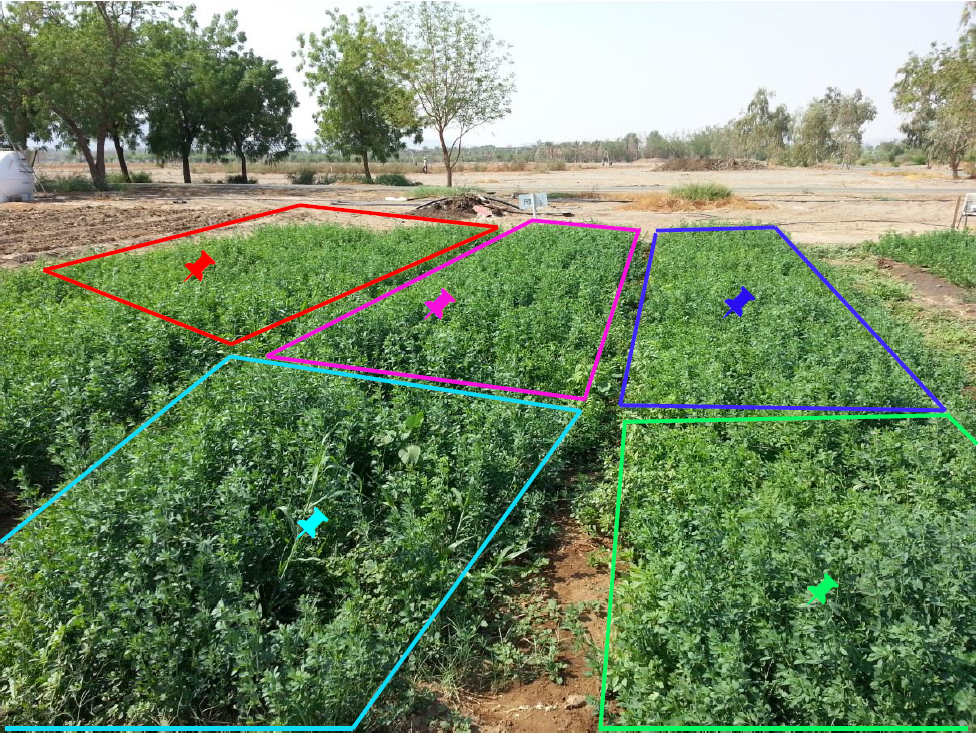

Supplement: Supplementary file 1 [file Image1.png]
